# Unsuspected pulmonary alveolar proteinosis in a patient with acquired immunodeficiency syndrome: a case report

**DOI:** 10.1186/1752-1947-5-46

**Published:** 2011-02-01

**Authors:** Dimple Tejwani, Angel E DeLaCruz, Masooma Niazi, Gilda Diaz-Fuentes

**Affiliations:** 1 Division of Pulmonary Medicine, Bronx Lebanon Hospital Center, 1650 Grand Concourse, Bronx, NY 10457, USA; 2 Department of Pathology, Bronx Lebanon Hospital Center, 1650 Grand Concourse, Bronx, NY 10457, USA

## Abstract

**Introduction:**

Diffuse lung infiltrates are a common finding in patients with acquired immunodeficiency syndrome and causes range from infectious processes to malignancies or interstitial lung diseases. Pulmonary alveolar proteinosis is a rare pulmonary disorder rarely reported in patients infected with human immunodeficiency virus. Secondary pulmonary alveolar proteinosis is associated with conditions involving functional impairment or reduced numbers of alveolar macrophages. It can be caused by hematologic malignancies, inhalation of toxic dust, fumes or gases, infectious or pharmacologic immunosuppression, or lysinuric protein intolerance.

**Case presentation:**

A 42-year-old African American man infected with human immunodeficiency virus was admitted with chronic respiratory symptoms and diffuse pulmonary infiltrates. Chest computed tomography revealed bilateral spontaneous pneumothoraces, for which he required bilateral chest tubes. Initial laboratory investigations did not reveal any contributory conditions. Histological examination of a lung biopsy taken during video-assisted thoracoscopy showed pulmonary alveolar proteinosis concurrent with cytomegalovirus pneumonitis. After ganciclovir treatment, our patient showed radiologic and clinical improvement.

**Conclusion:**

The differential diagnosis for patients with immunosuppression and lung infiltrates requires extensive investigations. As pulmonary alveolar proteinosis is rare, the diagnosis can be easily missed. Our case highlights the importance of invasive investigations and histology in the management of patients infected with human immunodeficiency virus and pulmonary disease who do not respond to empiric therapy.

## Introduction

Pulmonary alveolar proteinosis (PAP) was first described in 1958 by Rosen *et al*.[1]. It is a rare pulmonary disorder characterized by an abnormal accumulation of surfactant-derived material in the alveoli, leading to disease that ranges from mild symptoms with complete spontaneous resolution to progressive disease with ensuing respiratory failure [1,2].

Associated infections have been reported in 5 to 20% of PAP cases. This wide range may be due to reporting bias or difficulties detecting infectious processes [3]. The infectious agents include *Nocardia asteroides*, *Mycobacterium tuberculosis*, *Mycobacterium avium-intracellulare, Pneumocystis jirovecii *(formerly *carinii*) and cytomegalovirus (CMV). Most of these infectious agents have been reported in immunocompromised patients uninfected with human immunodeficiency virus (HIV); however, PAP is a rare finding in patients with HIV [4].

## Case presentation

We present a unusual case of a patient with HIV infection admitted with chronic respiratory symptoms, diffuse pulmonary infiltrates and bilateral spontaneous pneumothoraces, who was found to have PAP concurrent with *Cytomegalovirus pneumonitis*. Our patient was a 42-year-old African-American man, who was admitted with fever, productive cough with whitish sputum, fatigue, and weight loss of 6.8 kg in the previous month. He reported no visual abnormalities, and denied traveling or relevant any medical or surgical history.

Our patient performed maintenance work, and smoked 20 packs of cigarettes per year, but denied alcohol or substance use. He had undergone testing for tuberculosis (purified protein derivative) in the previous year, which was negative.

**Figure 1:**
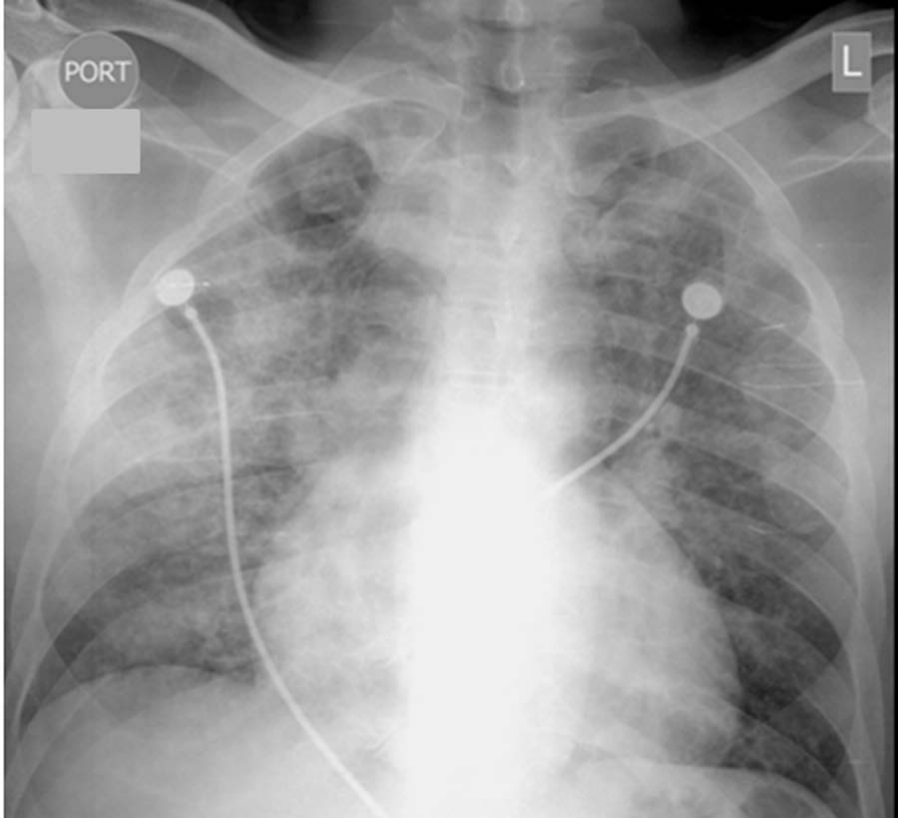
**Chest radiograph showing bilateral interstitial-alveolar pattern**.

**Figure 2:**
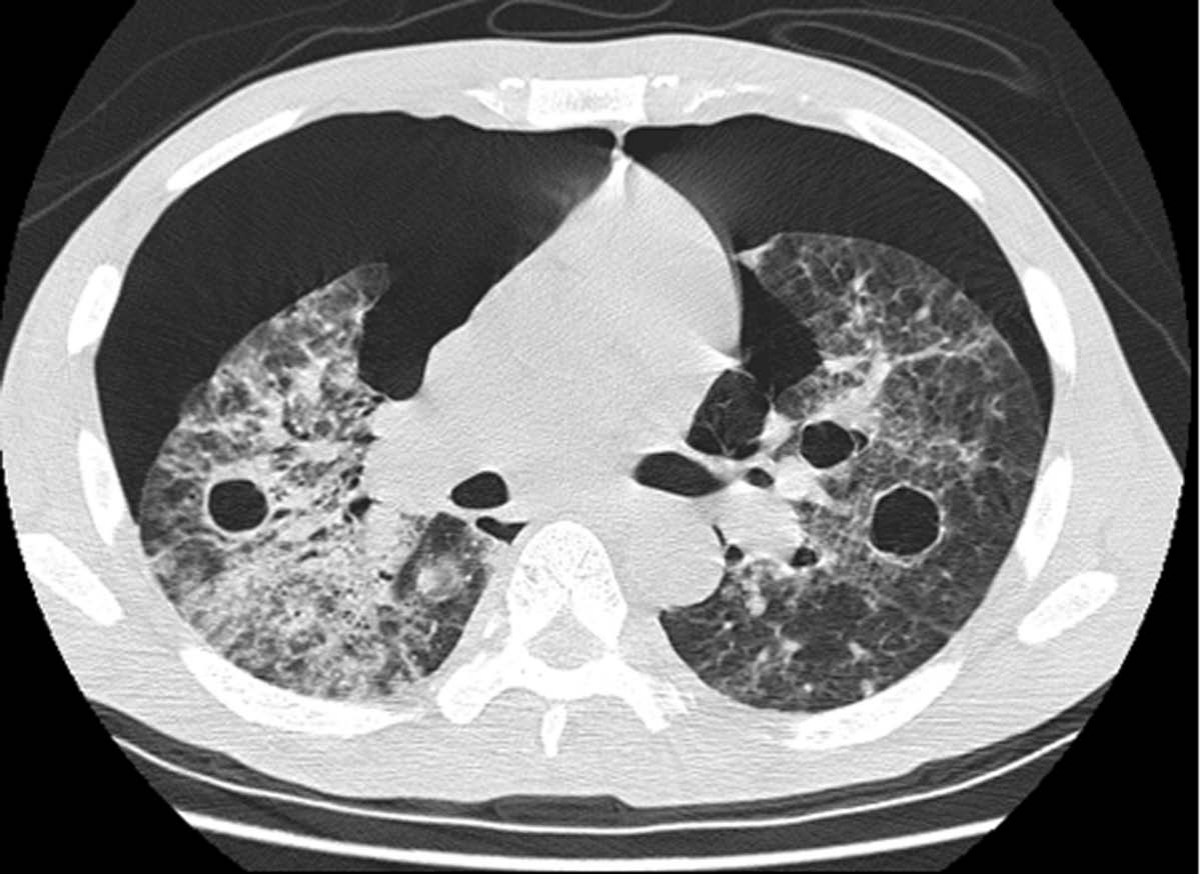
**Chest computed tomography scan showing bilateral pneumothoraces, several pneumatoceles, bilateral airspace consolidation, and ground-glass opacity**.

On physical examination, our patient was found to be febrile, tachycardic and tachypneic. His lungs were clear on auscultation, and the rest of the examination was normal. Arterial blood gas analysis revealed PaO_2 _of 79 mm Hg (80-100 mmHg normal range at ambient air) and SaO_2 _of 93% in 2 L of oxygen (normal values are 97% to 99% at ambient air). Initial laboratory test results showed elevated lactate dehydrogenase (LDH) of 528 U/L, normal liver and kidney function, and packed cell volume of 32%. Results for HIV testing were positive, with a CD4+ T-cell count of 12 cells/μL. Chest radiography showed a bilateral interstitial pattern (Figure 1). Chest computed tomography (CT) revealed bilateral pneumothoraces, multiple pneumatoceles, and bilateral consolidation with ground-glass opacity (GGO) (Figure 2).

Because of progressive dyspnea, our patient was transferred to the intensive care unit 24 hours after admission, where he underwent bilateral chest tube insertion. The initial differential diagnosis was community-acquired pneumonia or an opportunistic infection, typically *Pneumocystis *in a patient infected with HIV. Pneumothoraces and elevated LDH supported the diagnosis of *Pneumocystis *pneumonia (PCP), therefore antibiotics (ceftriaxone, azithromycin, trimethoprim-sulfamethoxazole) and corticosteroids were initiated. Sputum studies for PCP, acid-fast bacilli (AFB) and influenza were negative, as were blood and urine cultures. Our patient refused fiberoptic bronchoscopy.

Our patient's condition continued to deteriorate despite treatment. On the third day after admission, he required noninvasive positive pressure ventilation (fraction of inspired oxygen was 50%) to maintain O_2 _saturation at 92%. Because of a persistent air leak, two chest tubes were required in each lung, and on day six after admission, our patient underwent bilateral sequential video-assisted thoracoscopic surgery and lung biopsy. Histological examination of the biopsy revealed foamy macrophages, and PAS-positive, diastase-resistant and mucicarmine-negative material. *Pneumocystis *organisms were not detected by direct immunofluorescence with monoclonal antibodies. Histopathology revealed CMV inclusion bodies and proteinaceous material filling the alveoli.

**Figure 3:**
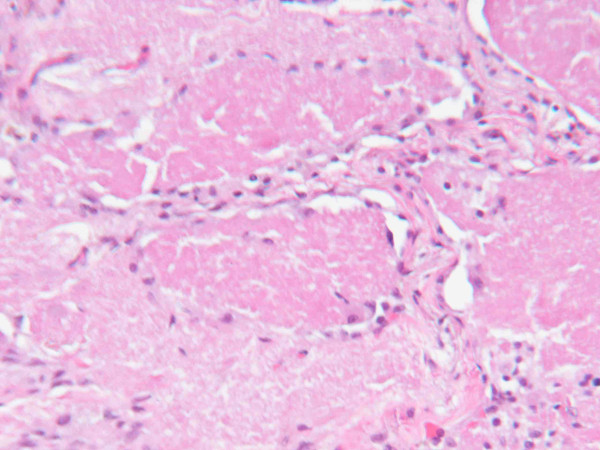
**Lung biopsy showing proteinaceous material filling the alveoli. Magnification × 200**.

**Figure 4:**
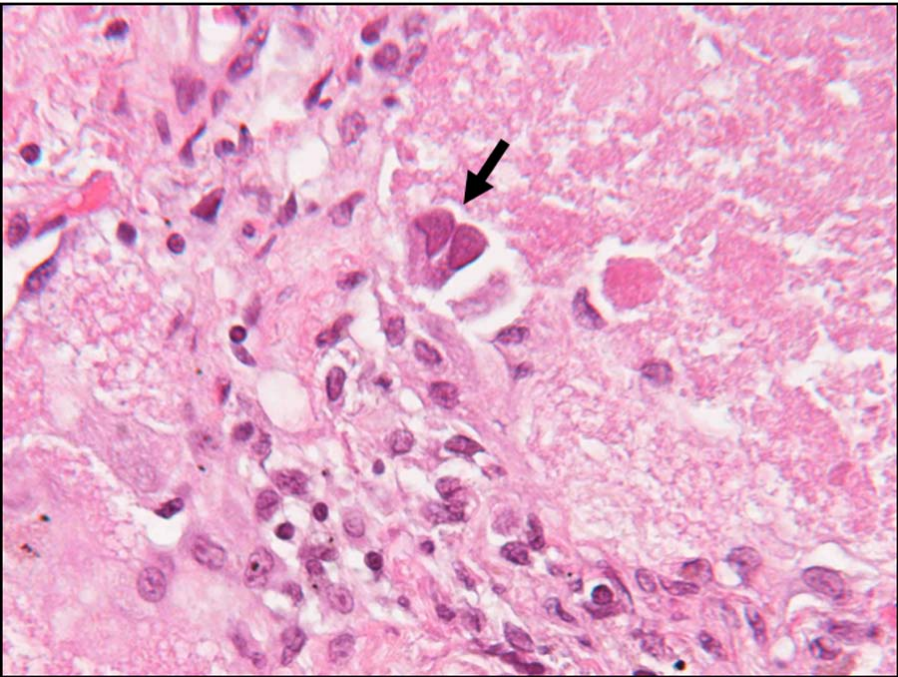
**Lung biopsy showing cytomegalovirus inclusion body (arrow) in a background of proteinaceous material filling the alveoli. Magnification × 400**.

**Figure 5:**
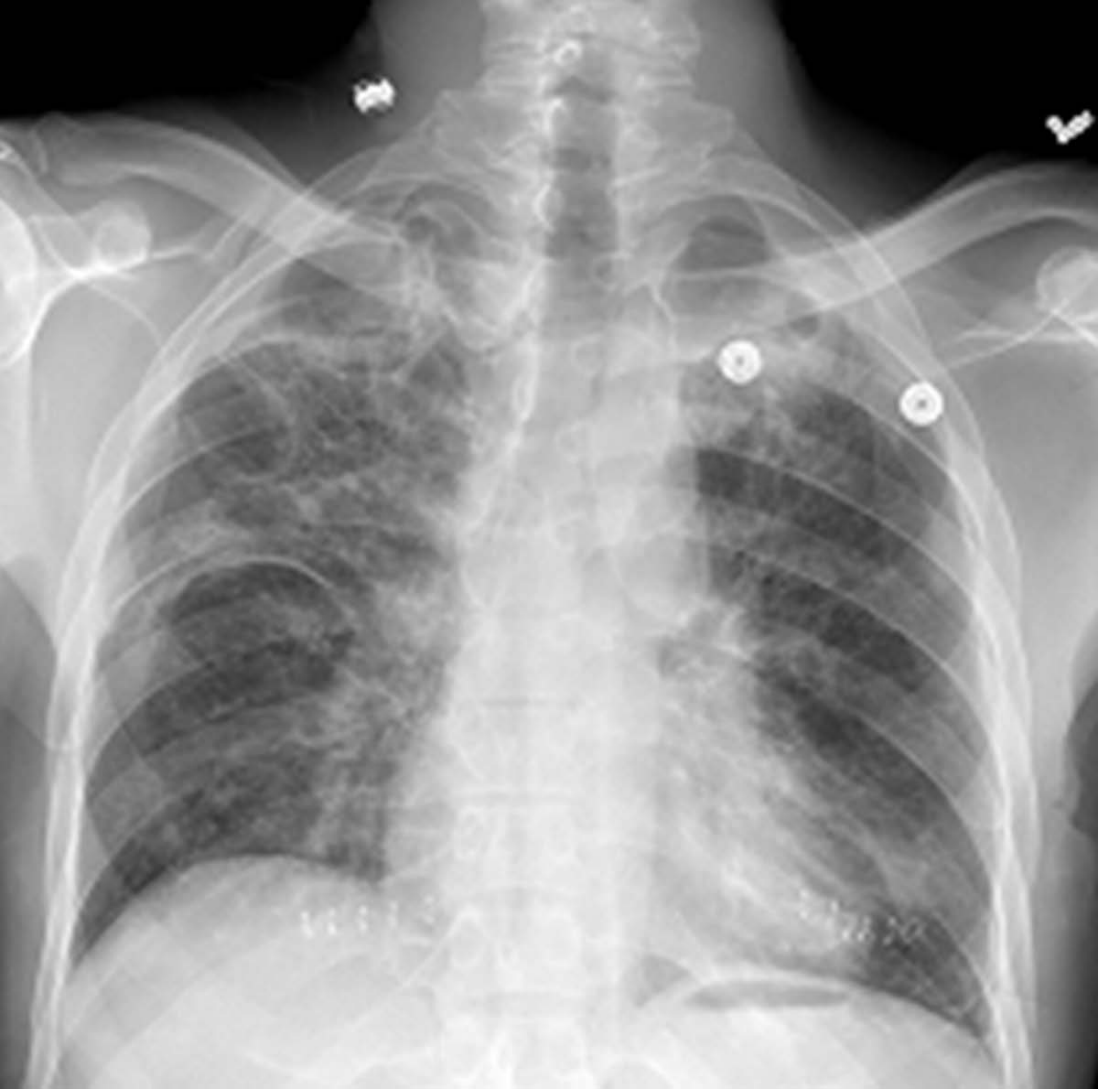
**Chest radiograph at discharge showing improvement in infiltrates**.

On day 10 after admission, ganciclovir was started, and the other antibiotics were discontinued (Figure 3, Figure 4). Results of serology testing for CMV were positive, and ophthalmology evaluation for CMV retinitis was negative. Our patient showed clinical and radiologic improvement, and he was discharged 46 days after admission (Figure 5).

## Discussion

Epidemiological data regarding the incidence and prevalence of PAP have been gathered from small case series and single case reports. The incidence of PAP is reported to be 0.36 to 0.49 cases per million in the population, with a prevalence of 3.70 to 6.2 cases per million. PAP occurs in all age groups, but is most common in men (male:female ratio 3:1) and among people aged 20 to 50 years. PAP is three times as common in smokers than in non-smokers, and in North America, 72% of patients with PAP are smokers [2,4].

There are three clinically distinct forms of PAP: congenital (2% of cases), acquired (also referred as primary or idiopathic, 90%), and secondary (5 to10%) [2].Congenital PAP is a heterogeneous collection of disorders caused by homozygous mutation of the genes encoding surfactant proteins (SP)-B and SP-C and the ABCA3 (ATP-binding cassette, sub-family A, member 3) transporter, or by the absence of the granulocyte macrophage colony-stimulating factor (GM-CSF) receptor [3,5]. Primary PAP is regarded as an autoimmune condition. It is characterized by excess surfactant caused by GM-CSF-neutralizing antibodies, receptor deficiency or gene deficiency/mutation, which leads to decreased macrophage stimulation. As a result, the immature alveolar macrophages are incapable of proper surfactant clearance [4-6]. Secondary PAP is uncommon, and develops in association with conditions involving functional impairment or reduced numbers of alveolar macrophages. It is caused by hematologic malignancies, inhalation of toxic dust, fumes or gases, infectious or pharmacologic immunosuppression, and lysinuric protein intolerance.

Patients infected with HIV have altered immunity and are susceptible to opportunistic lung infections. The subsequent breakdown of the alveolar lining, overproduction of substances normally secreted into the alveoli, impairment of alveolar clearance, and the transudation of plasma constituents into the alveoli may contribute to the pathogenesis of PAP [7]. Despite these risk factors, few reports exist of PAP in patients infected with HIV, and those cases of PAP that have been reported have been primarily associated with *P*. *jirovecii*, mycobacteria. or rarely, CMV infections [7-10].

The clinical presentation of PAP varies from asymptomatic (31% of acquired cases) to a more chronic presentation with dyspnea (39%), dyspnea and cough (11%), or cough only (10%). Cough is usually nonproductive, but is sometimes accompanied by sputum described as 'white and gummy' or 'chunky'. Fever and weight loss can also occur.

The physical examination is typically nonspecific: crackles, clubbing and cyanosis all have been reported, but rarely [11]. The radiographic findings are nonspecific, with chest radiography typically showing bilateral central and symmetric lung opacities with relative sparing of the apices and costophrenic angles, and less commonly, multifocal asymmetric opacities. Extensive diffuse consolidations have also been reported, suggesting interstitial pulmonary edema. Lymphadenopathy is rarely present. Chest CT findings are nonspecific and show smooth thickening of septal lines superimposed on areas of GGO, known as 'crazy paving'. A high-resolution CT study reported that secondary PAP was significantly more diffuse than autoimmune PAP. Pneumothoraces associated with PAP have been rarely reported, usually in association with PCP. In addition, a report suggests that emphysematous bullae in patients with PAP could lead to pneumothoraces [12-14].

Abnormal nonspecific laboratory findings in PAP include increased levels of serum LDH and other protein products of pulmonary epithelial cells such as carcinoembryonic antigen, cytokeratin 19, KL-6 mucin, SP-A, SP-B and SP-D [2]. GM-CSF auto-antibodies are elevated in primary PAP, but normal in secondary and congenital PAP [2,4]. Pulmonary function test usually reveals restrictive lung disease, decreased carbon monoxide diffusing capacity, increased alveolar-arterial partial oxygen pressure (PO_2_) gradient, hypoxemia and elevated shunt fraction.

The gold standard for PAP diagnosis is open lung biopsy, but fiberoptic bronchoscopy can diagnose up to 75% of PAP cases. Bronchoalveolar lavage and transbronchial biopsy are usually performed to exclude infection. The classic findings include a 'milky' fluid containing large amounts of granular acellular eosinophilic proteinaceous material, with morphologically abnormal 'foamy' macrophages engorged with diastase-resistant PAS-positive intracellular inclusions. Mucicarmine and PCP stains are negative, as in our case. When electron microscopy is available, the presence of concentrically laminated phospholipid structures called lamellar bodies can confirm the diagnosis [6,11,15]. Lung lavage fluid samples generally do not contain microbes, and it is now known that most cases of encountered infection are a secondary event rather than the initiating process [2].

Treatment of PAP depends on the physiologic impairment, rate of progression or remission and the underlying pathology. Supportive treatment and occasional lung transplantation are used for congenital PAP. Secondary PAP is managed with conservative therapy and treatment of the associated condition. In primary PAP, the standard of care is whole-lung lavage performed under general anesthesia. GM-CSF replacement is still experimental. The appropriateness of whole-lung lavage for secondary PAP with severe respiratory impairment is unclear at the present time [2,3,11].

## Conclusion

In conclusion, the differential diagnosis of diffuse lung infiltrates in patients with acquired immunodeficiency syndrome requires extensive investigation, as causes range from infectious processes to malignancies or interstitial lung diseases. Pneumothoraces in patients with HIV infection are usually attributed to PCP infection or emphysema caused by tobacco use or by the HIV infection itself. PAP is rare in these patients, thus it can easily be misdiagnosed. Clinicians caring for patients infected with HIV must consider PAP, either alone or in combination with CMV or other opportunistic infection, during the differential diagnosis. Tissue diagnosis is important, as is careful histological examination of bronchoscopic lavage fluid and biopsies. A surgical lung biopsy should be considered in cases of infiltrates of unclear etiology or progressive clinical deterioration despite treatment.

## Competing interests

The authors declare that they have no competing interests.

## Consent

Written informed consent was obtained from the patient for the publication of this case report and any accompanying images. A copy of the written consent is available for review by the Editor-in-Chief of this journal.

## Authors' contributions

DT, AED and GDF were responsible for the study conception, data retrieval and draft of the manuscript. MN selected, prepared and commented on the imaging. All authors read and approved the final manuscript.
